# First report on the physicochemical and proteomic characterization of *Proteus mirabilis* outer membrane vesicles under urine-mimicking growth conditions: comparative analysis with *Escherichia coli*

**DOI:** 10.3389/fmicb.2024.1493859

**Published:** 2024-11-06

**Authors:** María José González, Nicolás Navarro, Erlen Cruz, Sofía Sánchez, Javier O. Morales, Pablo Zunino, Luciana Robino, Analía Lima, Paola Scavone

**Affiliations:** ^1^Laboratorio de Biofilms Microbianos, Departamento de Microbiología, Instituto de Investigaciones Biológicas Clemente Estable, Montevideo, Uruguay; ^2^Drug Delivery Laboratory, Departamento de Ciencias y Tecnología Farmacéuticas, Universidad de Chile, Santiago, Chile; ^3^Advanced Center for Chronic Diseases (ACCDiS), Center of New Drugs for Hypertension and Heart Failure (CENDHY), Santiago, Chile; ^4^Departamento de Microbiología, Instituto de Investigaciones Biológicas Clemente Estable, Montevideo, Uruguay; ^5^Unidad Académica de Bacteriología y Virología, Instituto de Higiene, Facultad de Medicina, Universidad de la República, Montevideo, Uruguay; ^6^Unidad de Bioquímica y Proteómica Analíticas, Institut Pasteur de Montevideo & Instituto de Investigaciones Biológicas Clemente Estable, Montevideo, Uruguay

**Keywords:** outer membrane vesicles, urinary tract infections, uropathogenic *Escherichia coli*, *Proteus mirabilis*, proteomic, mass spectrometry

## Abstract

**Introduction:**

Uropathogenic bacteria employ multiple strategies to colonize the urinary tract, including biofilm formation, invasion of urothelial cells, and the production of adhesins, toxins, and siderophores. Among the most prevalent pathogens causing urinary tract infections (UTIs) are Uropathogenic *Escherichia coli* and *Proteus mirabilis*. A notable feature of Gram-negative bacteria is their ability to produce outer membrane vesicles (OMVs), which play critical roles in bacterial survival, virulence, and host-pathogen interactions, including UTIs.

**Methods:**

In this study, OMVs were isolated and characterized from two clinical strains, *E. coli* U144 and *P. mirabilis* 2,921, cultured in both Luria-Bertani broth and artificial urine.

**Result and discussion:**

The OMVs ranged in size from 85 to 260 nm, with the largest vesicles observed in artificial urine. Proteomic analysis allowed the identification of 282 proteins in OMVs from *E. coli* and 353 proteins from P. mirabilis when cultured LB medium, while 215 were identified from *E. coli* and 103 from *P. mirabilis* when cultured in artificial urine. The majority of these proteins originated from the bacterial envelope, while others were linked to motility and adhesion. Notably, the protein composition of OMVs varied depending on the growth medium, and proteins associated with zinc and iron uptake being more prominent in artificial urine, suggesting their importance in the urinary environment. Crucially, this is the first report to characterize *P. mirabilis* OMVs under different culture conditions, offering novel insights into the role of OMVs in UTI pathogenesis. These findings provide a deeper understanding of the molecular mechanisms by which OMVs contribute to bacterial virulence, establishing the foundation for potential therapeutic interventions targeting OMV-mediated processes in UTIs.

## 1 Introduction

Urinary tract infections (UTIs) are some of the most common bacterial infections that affect human beings (Stamm and Norrby, 2001). These infections are classically defined by the invasion and growth of bacteria in the urinary tract, accompanied by clinical symptoms. Among the most common etiological agents are uropathogenic *Escherichia coli* (UPEC) and *Proteus mirabilis,* which can cause both uncomplicated and complicated UTIs (Foxman, 2014). Those bacteria can survive in the urinary tract using several pathogenic mechanisms such as biofilm formation, uroepithelial cell invasion, adhesins, toxins, and siderophores (Hannan et al., 2012; Schaffer and Pearson, 2015). UPEC and *P. mirabilis* have different strategies for invading and persisting in the bladder. UPEC can invade the uroepithelial cell and form intracellular bacterial communities (IBC), providing the bacteria the ability to survive TLR4-mediated expulsion, cell exfoliation, urination, inflammation, and antimicrobial treatments (Rosen et al., 2007; Schwartz et al., 2011). On the other side, *P. mirabilis* produces MR/P pili that facilitate biofilm formation and colonization of the bladder and kidneys, being also crucial for catheter-associated biofilm formation (Armbruster and Mobley, 2012). The production of the urease enzyme provides a nitrogen source for *P. mirabilis*, and is highly important in pathogenesis (Milo et al., 2021). It is also important to consider that urine represents a moderately oxygenated environment, with high-osmolarity, and iron-limitation (Zunino et al., 1994).

Some of the main virulence mechanisms that allow these bacteria to colonize the urinary tract are flagella, fimbriae, and iron-uptake systems (Govindarajan and Kandaswamy, 2022). Flagella are mainly related to motility, contributing to bacterial spread during UTI, with biofilm formation (Zunino et al., 1994; Scavone et al., 2023), while improving bacterial fitness (Lane et al., 2005). Fimbriae play a crucial role in facilitating adhesion among bacteria, and to different abiotic and biotic surfaces, as uroepithelial cells (Mendoza-Barberá et al., 2023; Shanmugasundarasamy et al., 2022). In clinical UPEC strains, FimH adhesin (type I fimbriae) is widely distributed in pediatric patients presenting complicated UTIs, and interacts with uroplakin proteins in the bladder allowing entry into the eukaryotic cell (Luna-Pineda et al., 2016). In *P. mirabilis*, the adherence of the bacterium to epithelial and surfaces is mediated by 17 different fimbriae, among which MR/P fimbriae is the most prominent (Armbruster et al., 2018). As the urinary tract is iron-restricted, iron scavenging uptake systems are induced during infection (Himpsl et al., 2010). The iron scavenging systems play a crucial role in the bacteria growth and colonization; increasing iron concentrations could improve biofilm formation and enhance the bacterial resistance to antibiotics (Govindarajan et al., 2022). UPEC can acquire iron from the environment using three classes of systems: siderophores, hemophores (or heme binding and uptake systems), and direct ferrous iron (FeII)-uptake systems (Subashchandrabose and Mobley, 2015). *P. mirabilis* has developed 21 iron acquisition systems including siderophore-based mechanisms, ferrous iron transport, metal-type ABC transporters, among others (Chakkour et al., 2024).

Gram-negative bacteria naturally produce extracellular outer membrane vesicles (OMVs) (Kim et al., 2015), spherical bilayered particles released from the bacterial outer membrane, showing a diameter size ranging from 20 to 250 nm (Kulp and Kuehn, 2010). It has been also reported that OMVs might have different functions depending on their content and/or their target cells (Ellis and Kuehn, 2010). OMVs production has also been related to the bacterial stress response (McBroom and Kuehn, 2007). Bacterial OMVs can mediate antibiotic resistance, nutrient acquisition, and several bacteria-bacteria interactions, such as killing of competing bacteria (Lee et al., 2016). On the other hand, OMVs can participate in bacteria-host interactions, such as promoting inflammatory response, facilitating adhesion to eukaryotic cells, and delivering several virulence factors (Lee et al., 2016; Magaña et al., 2024; Charpentier et al., 2023).

Previously reported *E. coli* OMVs proteomic composition, showed that OMVs contain mainly outer membrane and periplasmic proteins (Lee et al., 2007). The presence of inner membrane proteins was low, suggesting that a specific sorting mechanism for vesicular proteins exists (Lee et al., 2007; Aguilera et al., 2014).

Despite the large amount of research regarding the potential functions of OMVs, their role in uropathogenic infections and their pathophysiological functions remain unclear. To further understand the role of OMVs in UTI, in this study, we carried out an extensive physicochemical characterization, and analyzed the total proteome of purified OMVs derived from UPEC and *P. mirabilis* strains obtained from cultures in Luria-Bertani broth and artificial urine. So far, this is the first proteomic characterization of *P. mirabilis* OMV.

## 2 Materials and methods

### 2.1 Bacterial strains and culture conditions

Two uropathogenic bacterial strains capable of forming biofilm were used in the present study: *Escherichia coli* U144 and *Proteus mirabilis* 2921. They were isolated from urine samples from patients with UTI and previously characterized in our laboratory (González et al., 2017; Zunino et al., 2000). Both strains were stored in glycerol at -80°C and recovered in Luria-Bertani agar (LA) at 37°C. The bacterial samples used in the present analyses were obtained previously and were anonymized.

### 2.2 Outer membrane vesicle isolation and purification

Outer membrane vesicles (OMVs) isolation and purification were performed as previously described (Tashiro et al., 2017). For OMV isolation, the bacterial strains were grown in Luria-Bertani (LB) broth and artificial urine (AU: in g/L; CaCl_2_, 0.49; MgCl_2_, 0.65; NaCl, 4.6; Na_2_SO_4_, 2.3; sodium citrate, 0.65; sodium oxalate, 0.02; KH_2_PO_4_, 2.8; KCl, 1.6; NH_4_Cl, 1.0; urea, 25; and trypticase soy broth; pH 6.2; Soriano et al., 2009). AU was sterilized using a Millipore Membrane 0.45 μm pore size. To obtain OMV samples without bacterial cells, 100 mL of overnight batch cultures were centrifuged at 6000×g, for 15 min, at 4°C. The supernatants were filtered through 0.45 and 0.20 μm membrane filters, and ultracentrifuged at 100000×g, for 2 h, at 4°C using a fixed-angle rotor (90 Ti, Beckman). The pellets were resuspended in 300 μL of 50 mM HEPES, 0.85% NaCl (HEPES-NaCl buffer). For OMV purification, samples were adjusted to 1 mL of 45% (w/v) iodixanol (OptiPrep™, Sigma) in HEPES-NaCl, transferred to the bottom of ultracentrifuge tubes, and layered with iodixanol-HEPES-NaCl (2 mL of 40, 35, 30, 25, and 20%). The samples were ultracentrifuged at 100,000 × g, for 3 h, at 4°C using a swing rotor (sw 40 Ti, Beckman). Then, several 1 mL fractions were collected from each gradient. To confirm the fraction containing OMVs, the protein concentrations in each fraction were measured using the Bradford protein assay and UV–Vis absorbance at 280 nm. The fraction containing OMVs was ultracentrifuged at 100,000 × g, for 2 h, at 4°C and resuspended in HEPES-NaCl.

### 2.3 Charaterization of OMV for dynamic light scattering measurements and laser doppler electrophoresis

The hydrodynamic radius and polydispersity index (PdI) were acquired by the dynamic light scattering technique (DLS), and the zeta potential was measured by laser Doppler electrophoresis in a Zetasizer ZS (Malvern Instruments), with a He-Ne laser light source at 633 nm with a fixed scattering angle of 175°. The OMV samples were diluted with Milli-Q water and were measured on a DTS1070 cuvette.

### 2.4 Nanoparticle tracking analysis

OMVs concentration was determined using the Nanoparticle Tracking Analysis (NTA). All the measurements were made in a Nanosight NS300 (Malvern Instruments) in triplicate at 25°C. The light dispersion was captured by a sCMOS camera. The samples were diluted 1/100 in filtered Milli-Q water. The obtained hydrodynamic radius, concentration, and standard deviation correspond to the arithmetic values for all the particles analyzed on the sample by the NTA software.

### 2.5 Transmission electron microscopy

For Transmission electron microscopy (TEM) analysis of OMVs, the samples were diluted and deposited onto a copper grid, stained with 1% phosphotungstic acid, dried at room temperature, and visualized at 10.00 kV using an Inspect F50 scanning transmission electron microscope (FEI Company, Facultad de Ciencias Químicas y Farmacéuticas, Universidad de Chile). The OMVs size measurements were obtained as the average size from 10 microimages for each condition.

### 2.6 OMVs protein analysis by SDS-PAGE

OMVs protein concentration was determined by densitometry analysis of Colloidal Coomassie stained gels. For that purpose, each sample was loaded into pre-cast gels (NuPAGE™ 4–12%, Bis-Tris, 1.0 mm, Mini Protein Gel, 10-well, Invitrogen), using LMW-SDS Marker Kit (Cytiva). Electrophoresis was run at 150 V. Gels were fixed with 50% ethanol and 10% acetic acid and stained overnight with colloidal Coomassie blue. After destaining with ultrapure water washing, gel images were digitalized with UMAX Power-Look 1,120 scanner and LabScan 5.0 software (GE Healthcare). Quantification was performed using the ImageQuant TL software (v8.1), 1D analysis module, and LMW-SDS Marker Kit (Cytiva) as standard.

### 2.7 Preparation of OMV proteins for mass spectrometry analysis

Proteomic analysis of OMVs obtained from *E. coli* and *P. mirabilis* grown in LB broth and AU was carried out using three independent biological replicates. Twenty-five μL of each sample were loaded into a 12.5% acrylamide gel and run until samples enter 1 cm into the resolving gel. The gel was fixed and stained as described above. Gel fragments were sliced with a scalpel and transferred to microcentrifuge tubes. Sample processing for mass spectrometry analysis was performed as described previously (Rossello et al., 2017; Gil et al., 2019). Briefly, the procedure consisted in sample reduction with 10 mM DTT at 56°C for 1 h with vigorous agitation; cysteine alkylation with 50 mM iodoacetamide at room temperature for 45 min with vigorous agitation and protected from light; *in gel* protein digestion with 0.1 μg/μL trypsin (sequence grade, Promega) in 50 mM ammonium bicarbonate, overnight at 37°C; peptide extraction by incubation with 60% acetonitrile/0.1% trifluoroacetic acid with vigorous agitation for 2 h at 30°C; vacuum drying concentration and resuspension in 0.1% trifluoroacetic acid. Samples were desalted using C18 micro-columns (C18 OMIX pipette tips, Agilent), eluted with 0.1% formic acid in ACN, vacuum dried, and resuspended in 0.1% formic acid. Peptide samples concentration were determined using a Denovix DS-11 FX+ spectrophotometer/fluorometer, at 215 nm. Final volume was adjusted in order to normalize all sample concentrations.

### 2.8 Nano-liquid chromatography and mass spectrometry analysis

OMV protein samples were analyzed by nano-LC MS/MS using a shotgun strategy on a nano-HPLC, UltiMate 3,000 coupled on line to an Orbitrap Exploris 240 mass spectrometer through an Easy-Spray source. One ug of tryptic peptides was injected into an Acclaim PepMap™ 100 C18 nano-trap column (75 μm x 2 cm, 3 μm particle size), and separated using a 75 μm x 50 cm, Easy-Spray™ analytical C18 HPLC column (2 μm particle size, 100 Å pore size) at a constant flow rate of 200 nL/min and 40°C. The column was equilibrated at 1% of mobile phase B (A: 0.1% formic acid; B: 0.1% formic acid in acetonitrile) followed by an elution gradient from 1 to 35% B over 90 min and 35–99% B over 20 min. All nano-LC MS/MS equipment and supplies were from Thermo Scientific.

The mass spectrometer was operated in the positive mode. Ion spray voltage was set at 2.2 kV; capillary temperature at 250°C, and S-lens RF level at 50. Mass analysis was carried out in data dependent mode in two steps: acquisition of full MS scans in a range of *m/z* from 200 to 2000; followed by HCD fragmentation of the 20 most intense ions in each segment using a stepped normalized collision energy of 25, 30 and 35. Full MS scans were acquired at a resolution of 90,000 ppm at 200 *m/z*, and a maximum ion injection time of 100 ms. For MS/MS acquisition the resolution was 22,500 ppm, and maximum ion injection time: 50 ms. Precursor ions with charge states from 2 to 5 were included for fragmentation. Dynamic exclusion time was set at 30 s.

### 2.9 Protein identification and analysis

Database generation, protein identification, and analyses were performed using the PatternLab V software (Santos et al., 2022).^1^ Protein sequences from uropathogenic *E. coli* CFT073 and *P. mirabilis* HI4320 were downloaded from Uniprot Consortium (in December, 2023)^2^ and protein sequences of the 127 most common mass spectrometry contaminants were used to generate target-decoy databases. Raw files were queried against each corresponding database using the Comet search engine (integrated in PatternLab V) applying the following parameters: fully specific trypsin as proteolytic enzyme with up to 2 missed cleavages allowed; methionine oxidation as variable modification and cysteine carbamidomethylation as fixed modification; 40 ppm tolerance from the measured precursor *m/z*.

Peptide spectrum matches were filtered using the Search Engine Processor (SEPro), setting the acceptable FDR criteria as follows: 3% at spectrum level, 2% at peptide level, and 1% at the protein level. PatternLab’s Approximately Area-Proportional Venn Diagram module was used to identify proteins exclusively detected in each sample set from each bacterium using a *p*-value ≤0.05. The Pairwise Comparison module was used to detect proteins present in both conditions but at significantly different relative abundance by means of extracted ion chromatogram (XIC) analysis. The following statistical criteria were applied: log2 fold change ≤1, and *p*-value ≤0.05.

In order to evaluate the surface-associated protein fraction in OMVs, the subcellular origin of identified proteins was analyzed *in silico* using the PSORTb v3.0 algorithm (Yu et al., 2010).

## 3 Results

### 3.1 Characterization of OMVs of *Escherichia coli* and *Proteus mirabilis*

**Figure 1:**
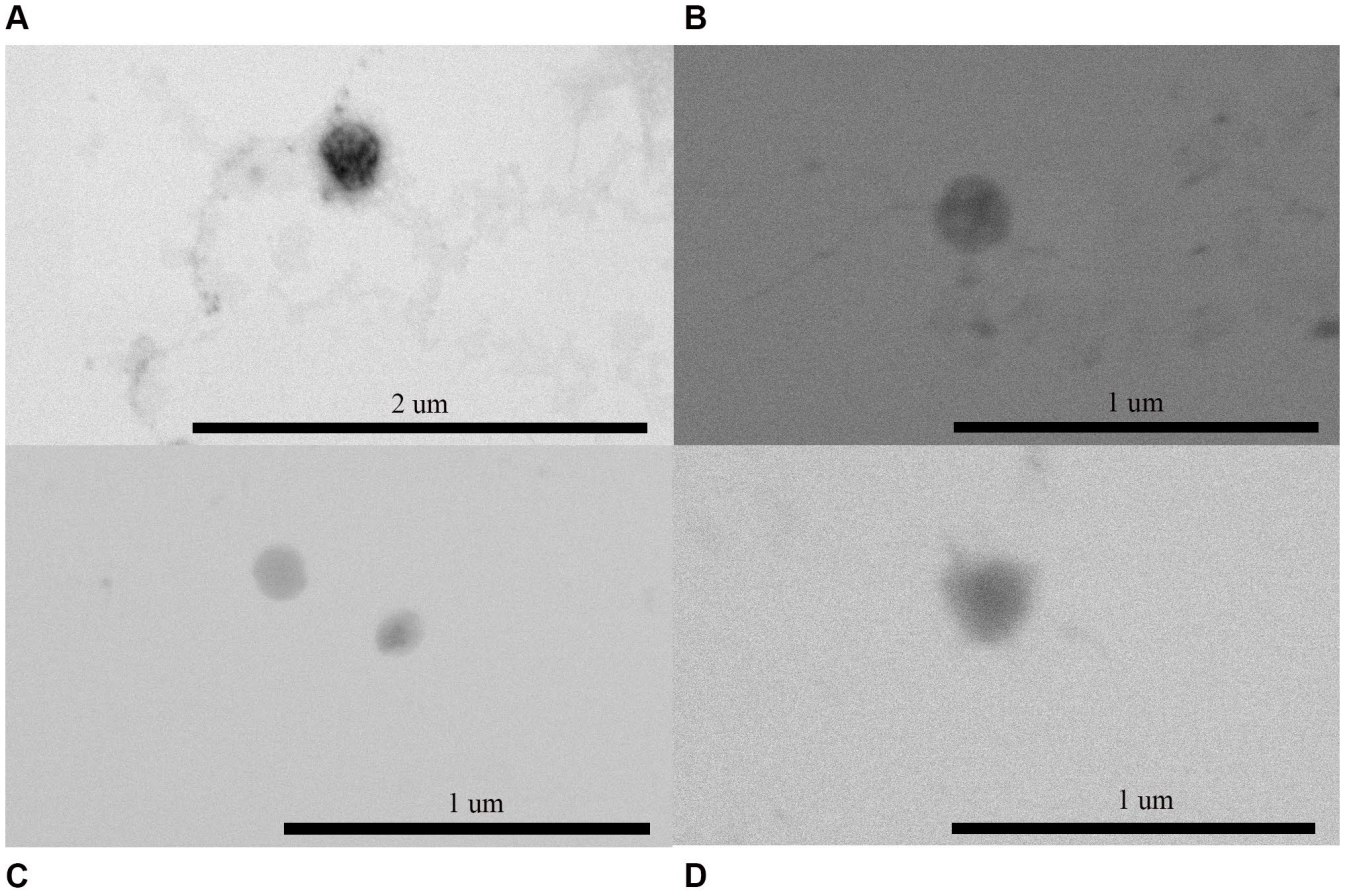
Visualization of OMVs isolated from (A) *Escherichia coli* in LB, (B) *E. coli* in AU, (C) *Proteus mirabilis* in LB broth, and (D) *P. mirabilis* in AU. OMVs were fixed and negatively stained and viewed under STEM (Facultad de Ciencias Químicas y Farmacéuticas, Universidad de Chile) showing a spherical shape. The scale bar indicated in (A) 2 μm, and in (B–D) 1 μm.

OMVs secreted by *E. coli* and *P. mirabilis* were isolated and then examined using scanning transmission electron microscopy (TEM) and dynamic light scattering (DLS). *E. coli* and *P. mirabilis* produced spherical OMV in both LB broth and AU with a size ranging from 85 to 260 nm, as shown in the TEM images (Figure 1). On the other hand, the sizes determined by DLS of *P. mirabilis* and *E. coli* OMVs obtained from cultures in LB were 267.6 ± 29.7 nm and 185 ± 25.9 nm, respectively. In AU, the sizes observed were 320.4 ± 32.2 and 257.6 ± 3.8 nm for *P. mirabilis* and *E. coli*, respectively. These measurements allowed us to observe that OMVs size recovered from bacteria grown in AU were larger compared to those grown in LB, for both strains. For bacteria grown in LB, the polydispersity factor was higher in *P. mirabilis* showing a value of 0.355, while in *E. coli* OMVs it was 0.263. In bacteria grown in AU, we also observed higher polydispersity factors in *P. mirabilis* OMVs compared to those obtained from *E. coli*, showing values of 0.344 and 0.214, respectively. Furthermore, these data indicated that all preparations presented low polydispersity (Table 1).

**Table 1:** Physicochemical characterization parameter of *Escherichia coli* and *Proteus mirabilis* OMVs.

	EcLB	EcAU	PmLB	PmAU
Size (DLS, d.nm)	185.5 ± 25.9	257.6 ± 3.80	267.6 ± 29.7	320.4 ± 32.2
Z potential (mV)	−36.5 ± 1.3	−7.5 ± 1.7	−37.4 ± 1.5	−15.1 ± 0.5
PdI	0.263	0.219	0.355	0.344
Concentration (NTA, particle/ml)	1.28E+11	1.12E+11	7.61E+10	6.67E+10

EcLB, OMVs obtained from LB. EcAU, *E. coli* OMVs obtained from AU. PmLB, *P. mirabilis* OMVs from obtained from LB. PmAU, *P. mirabilis* OMVs obtained from AU. DLS, Dynamic light scattering. PdI, Polydispersity index. NTA, Nanoparticle Tracking Analysis.

The zeta potentials recorded were -37.4 ± 1.5 mV and -36.5 ± 1.3 mV in LB, and -15.5 ± 0.5 mV and -7.5 ± 1.7 mV in AU, for *P. mirabilis* and *E. coli* OMVs, respectively. These values suggest very minor differences in OMVs stability between the two growth conditions.

The OMVs concentration was measured using NTA, evidencing concentrations of approximately 1×10^11^ particles/ml and 7×10^10^ particles/ml for *E. coli* and *P. mirabilis,* respectively. This assay showed that the amount of particles for each bacteria were similar in LB and AU (Table 1).

**Figure 2:**
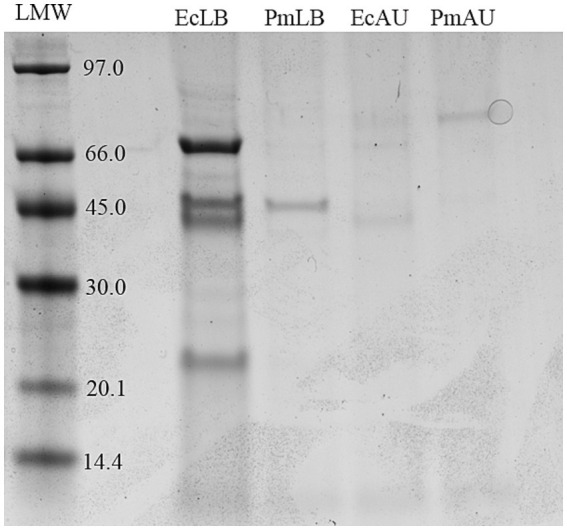
Protein profiles of OMV from *E. coli* in LB and AU (EcLB and EcAU), and *P. mirabilis* in LB and AU (PmLB and PmAU) run in 12% SDS-PAGE. Molecular weight standard is indicated on the left (kDa).

To observe the OMVs protein profiles, the vesicles were subjected to SDS-PAGE stained with Coomassie blue (Figure 2). Equal amounts of OMVs were loaded in order to compare their protein content. Comparison of OMVs preparations showed different protein profiles and protein yields among strains and media conditions.

### 3.2 Proteomic characterization of *Proteus mirabilis* OMV in LB and AU

LC–MS/MS analysis identified 353 proteins of OMVs from *P. mirabilis* grown in LB broth (PmLB), and 103 proteins from *P. mirabilis* OMVs cultured in AU (PmAU), considering those proteins that were statistically detected in at least two of the three replicates.

**Figure 3:**
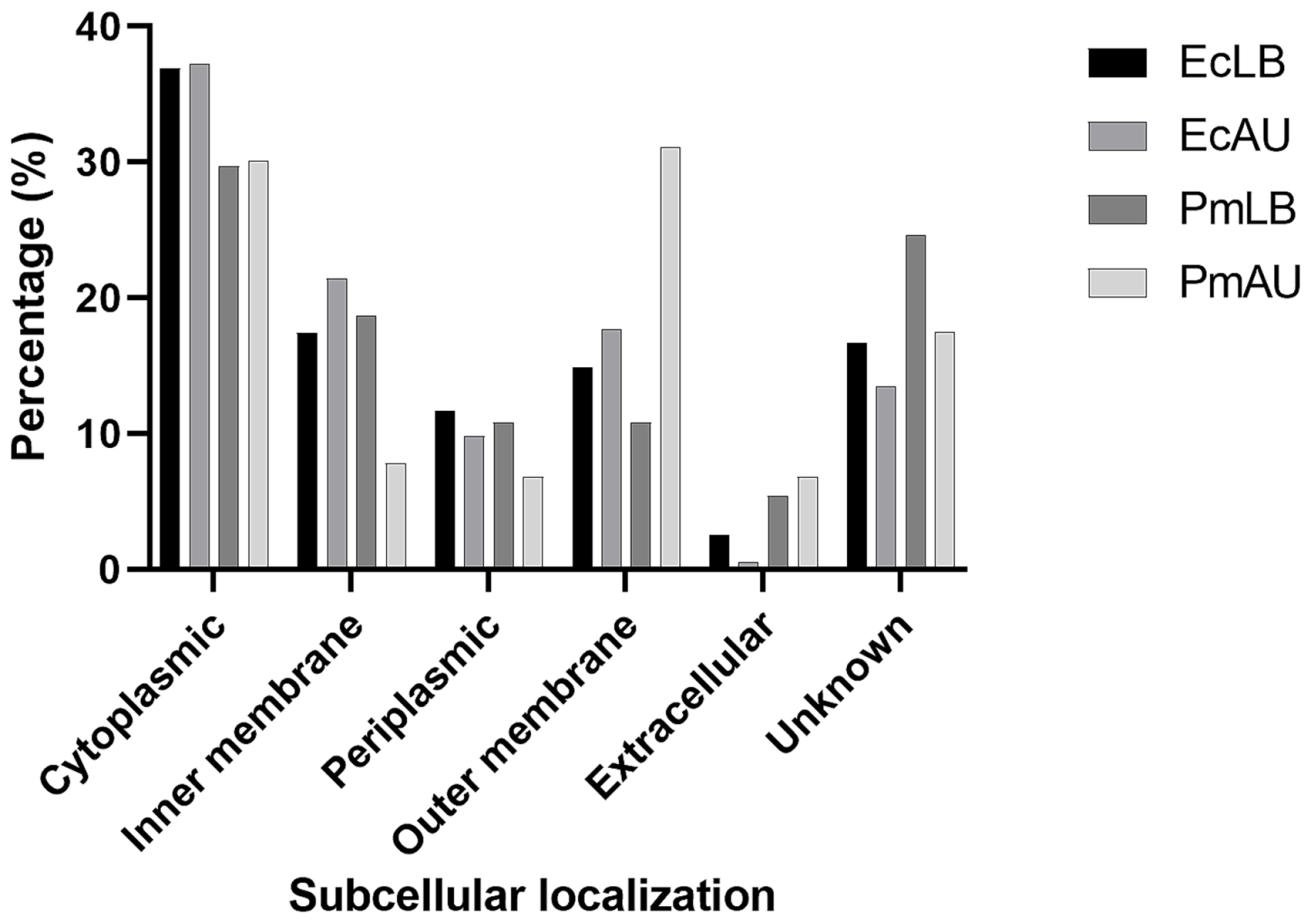
Distribution of subcellular localizations of vesicular proteins from *E. coli* and *P. mirabilis* in LB (EcLB and PmLB), and in AU (EcAU and PmAU), according to the PSORTb v3.0 algorithm.

The analysis of subcellular localization of the 353 identified proteins from *P. mirabilis* OMVs obtained in LB media showed the following classification: 105 (29.7%) cytoplasmic, 87 (24.6%) unknown, 66 (18.7%) inner membrane, 38 (10.8%) periplasmic, 38 (10.8%) outer membrane, and 19 (5.4%) extracellular (Figure 3).

On the other hand, the subcellular localization of the identified proteins from *P. mirabilis* OMVs cultured in AU showed the following distribution: 32 (31.1%) outer membrane, 31 (30.1%) cytoplasmic, 8 (7.8%) inner membrane, 7 (6.8%) periplasmic, and 7 (6.8%) extracellular. Eighteen identified proteins (17.5%) could not be classified into any of the above categories (Figure 3). Based on these analyses, we could observe that the percentage of inner membrane proteins was lower in AU than in LB OMVs. The opposite trend was found for the percentage of outer membrane proteins.

Among the most abundant proteins determined according to spectrum count in both culture conditions, we found some reported vesicle markers including OmpA, OmpF, OmpW, TolC, TolB, AcrA, TufB, Pal, and Lpp. Furthermore, several outer membrane proteins, such as TonB-dependent receptor (IreA), YaeT, MipA, NlpD, and FadL, were found to be highly abundant in this dataset. Also, the hemolysin HpmA was found (Supplementary Table 1).

### 3.3 Comparative analysis of *Proteus mirabilis* OMV in LB and AU

**Figure 4:**
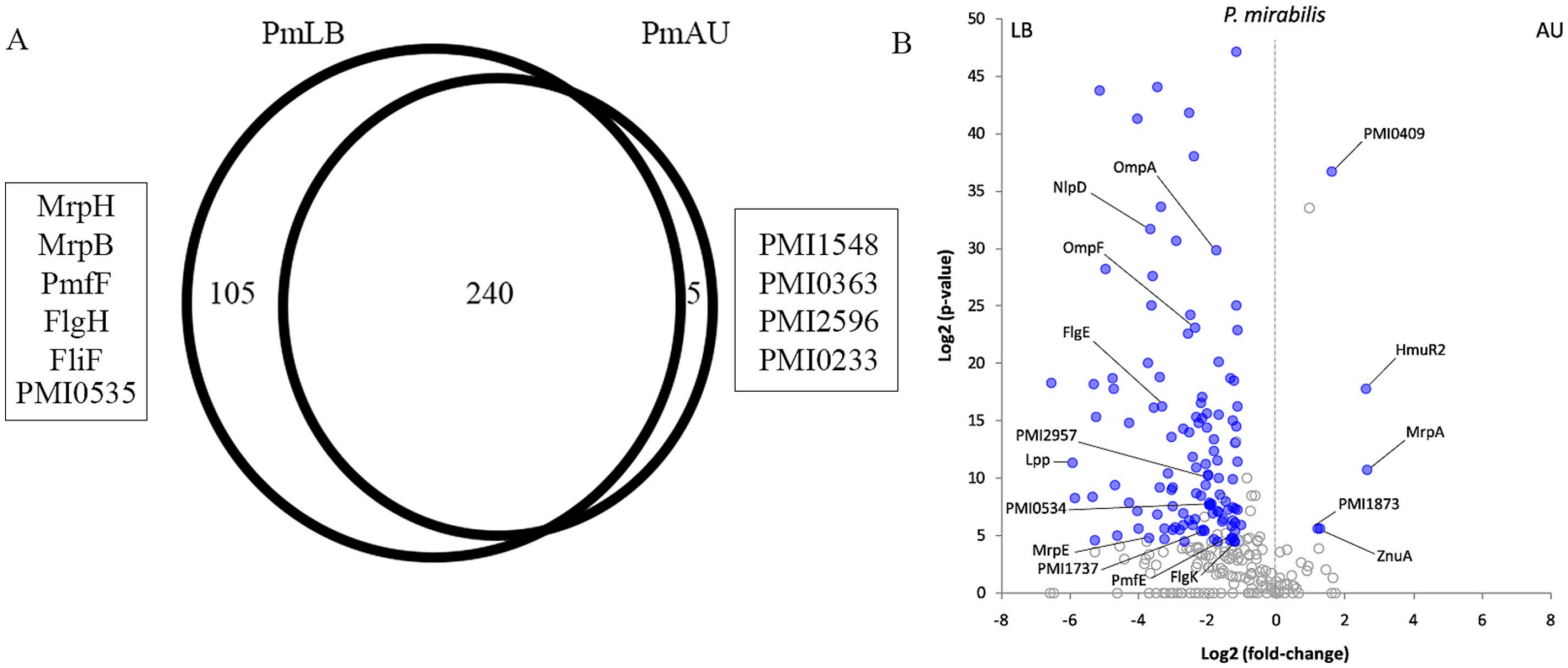
(A) Venn diagram of OMVs proteins overlapping between EcLB and EcAU, using the Venn diagram’s statistical module from the PatternLab V software. 271 proteins were shared between OMVs from both media, 47 proteins were exclusive of EcLB OMVs, and 14 proteins from EcAU OMVs. The proteins mentioned in the text are shown in the picture. (B) Differentially abundant proteins between *E. coli* OMVs obtained from AU and LB. The volcano plot shows the Log2 (*p*-value) on the y-axis and the Log2 (fold-change) on the x-axis. Proteins identified as common to both conditions are represented by a dot in the plot. Blue dots indicate proteins satisfying all statistical criteria and thus are considered as differentially abundant among culture conditions. Selected differential proteins discussed in the text are labeled. Information regarding all differential proteins are depicted in Supplementary Table 3.

Proteins identified in *P. mirabilis* OMVs obtained from LB broth were compared to those from AU culture. These analyses allowed us to determine that 105 proteins were exclusively detected in LB broth and only 5 proteins in AU (*p*-value <0.05; Figure 4A; Supplementary Table 2).

Using the PatternLab V Pairwise comparison module, we identified 118 and 5 proteins significantly increased in PmLB and PmAU, respectively (*p*-value <0.05; Figure 4B; Supplementary Table 2). Taking into account that the protein dataset obtained from *P. mirabilis* OMVs grown in LB was considerably larger than the one obtained in the same bacterium but grown in AU, and considering that this difference may lead to some bias and potential false positive, we proceed to comparatively analyze proteomic profiles of *E. coli* grown on the same conditions. Therefore, we focused our discussion on proteins supported by the *E. coli* OMVs comparative analysis and corroborated by evidence reported in the literature.

Within the proteins that were detected only in PmLB OMVs we found several fimbrial proteins including MrpH, PmfF, MrpB, PMI0535, and the flagellar proteins FlgH and FliF. Also, many other members of this family proteins were increased in the OMVs from LB broth such as MrpE, PMI0534, PmfE, FlgE and FlgK. Within OMVs from *P. mirabilis* grown in LB broth, we could observe several virulence factors related to adhesion and motility. The only fimbrial protein that was increased in OMVs from AU was MrpA (Table 2).

**Table 2:** Selected proteins identified in OMVs from *P. mirabilis* discussed in the text.

Accession (uniprot)	Protein	Description/annotation	Fold-change	*p*-value	Media condition	Subcellular location*
Flagellar proteins						
B4EYL5	FliF	Flagellar M-ring protein	ED	*p* < 0.05	LB	IM
B4EYN3	FlgH	Flagellar L-ring protein	ED	*p* < 0.05	LB	OM
B4EYN0	FlgK	Flagellar hook-associated protein 1	1.25	0.037	LB	E
B4EYN6	FlgE	Flagellar hook protein	3.30	1.3E-05	LB	E
Fimbrial proteins						
B4EUK6	MrpH	Fimbrial adhesin	ED	*p* < 0.05	LB	U
P53521	PmfF	Putative minor fimbrial subunit	ED	*p* < 0.05	LB	E
B4EUK0	MrpB	Fimbrial subunit	ED	*p* < 0.05	LB	E
B4EV67	PMI0535	Fimbrial chaperone	ED	*p* < 0.05	LB	P
B4EUK3	MrpE	Fimbrial subunit	3.99	0.021	LB	E
B4EV66	PMI0534	Fimbrial usher protein	1.94	0.005	LB	OM
P53522	PmfE	Putative minor fimbrial subunit	1.33	0.041	LB	E
Q03011	MrpA	Major MR/P fimbria protein	2.66	0.001	AU	E
Porins proteins						
B4EVB8	OmpF	Outer membrane porin	2.36	1.1E-07	LB	OM
B4EVD6	OmpA	Outer membrane protein A	1.75	1.0E-09	LB	OM
Lipoproteins						
B4EWN9	Lpp	Major outer membrane lipoprotein	5.91	3.9E-04	LB	OM
B4F222	NlpD	Lipoprotein	3.66	3.0E-10	LB	OM
B4EZ34	PMI1737	Lipoprotein	2.19	0.003	LB	U
B4EWL9	SlyB	Outer membrane lipoprotein	1.46	0.004	LB	OM
B4EZW1	PMI1873	Lipoprotein	1,21	0.021	AU	U
Transport proteins						
B4EZW8	PMI2957	Iron ABC transporter, substrate-binding protein	1.96	0.001	LB	IM
B4EY62	PMI1548	TonB-dependent receptor	ED	*p* < 0.05	AU	OM
B4EUU5	PMI0363	TonB-dependent ferric siderephore receptor	ED	*p* < 0.05	AU	OM
B4EXJ5	PMI2596	Siderophore TonB-dependent receptor	ED	*p* < 0.05	AU	OM
B4EUG9	PMI0233	TonB-dependent siderophore receptor	ED	*p* < 0.05	AU	OM
B4EUZ0	PMI0409	TonB-dependent receptor	1.62	9.0E-12	AU	OM
B4EX61	HmuR2	Hemin receptor	2.62	4.5E-06	AU	OM
B4EVT9	ZnuA	High-affinity zinc uptake system protein	1.30	0.020	AU	P

ED, Proteins exclusively detected (*p*-value < 0.05). The media condition are LB, Luria Bertani Broth; AU: Artificial urine. The subcellular locations are IM, Inner membrane; OM, Outer membrane; E, Extracellular; U, Unknown; P, Periplasmic. *The subcellular localization prediction by Psortb v3.

Several lipoproteins are shared between OMVs obtained in both culture conditions (LB broth and AU), but two of them (NlpD and SlyB) were increased in LB broth as shown in Table 2. The porin OmpA was more abundant in OMVs from PmLB as also in OMVs from EcLB.

The proteins present exclusively in OMVs from AU are most related with iron acquisition and belong to the TonB-dependent receptor family: PMI1448, PMI0363, PMI2596, and PMI0233. Also, PMI0409 was more abundant in AU. Other transporters showing significant increased relative abundance in OMVs from PmAU are ZnuA, related with zinc uptake, and HmuR2, an hemin receptor involved in Zinc and iron-acquisition.

### 3.4 Proteomic characterization of *Escherichia coli* OMV in LB and AU

LC–MS/MS analysis allowed the identification of 282 proteins from LB broth-grown *E. coli* OMVs and 215 proteins from AU-grown *E. coli* OMVs, considering those proteins that are statistically detected in at least two of the three replicates.

The analysis of subcellular localizations of the identified proteins from *E. coli* OMVs obtained in LB broth showed that the majority of them were from the bacterial envelope, including 49 (17.4%) from the inner membrane, 42 (14.9%) from the outer membrane, and 33 (11.7%) were classified as periplasmic proteins (Figure 3). On the other hand, 36.9% of total proteins (104) were classified as cytoplasmic, and 2.5% (7) extracellular. The subcellular localization of 46 proteins (16.6%) was classified as unknown (Figure 3).

A similar distribution of subcellular localization was observed in *E. coli* OMVs obtained in AU. Most of the proteins were from the bacterial envelope, including 46 (21.4%) from inner membrane, 38 (17.7%) from outer membrane, and 21 (9.8%) from the periplasm. Eighty of the identified proteins (37.2%) were cytoplasmic and only one was extracellular. Twenty-nine of identified proteins (13.5%) showed an unknown localization classification (Figure 3). From these analyses, it becomes apparent that, despite the similarities observed, there are slight differences in the percentage of cell envelope components, primarily in the inner and outer membranes in OMVs obtained from AU compared with those obtained from LB broth.

Among all the proteins identified in UPEC grown in both conditions, conserved outer membrane protein components were the most abundant as determined by spectrum count. Mainly, the outer membrane porin OmpA, OmpC, OmpF, OmpX, NmpC, and Maltoporine, exhibited the highest abundance. These porins were commonly observed as vesicle markers. Also, other outer membrane components considered as vesicles markers were found, including TolB, TolC, and AcrA. Additional proteins detected in OMVs from both media conditions, such as Lpp, Pal, Tsx, FhuA, and LamB, were previously reported in OMVs (Lee et al., 2007; Supplementary Table 2).

### 3.5 Quantitative comparative analysis of *Escherichia coli* OMV in LB and AU

**Figure 5:**
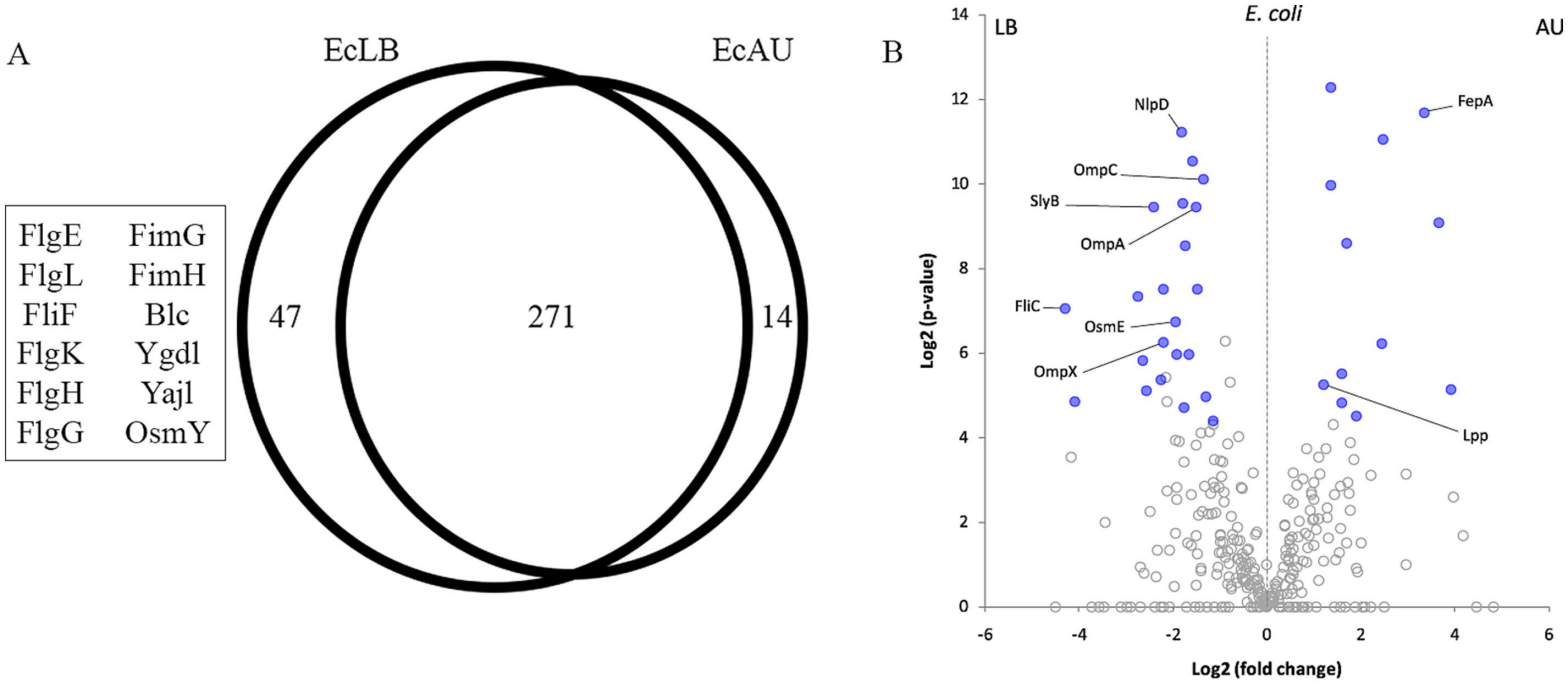
(A) Venn diagram of OMVs proteins overlapping between PmLB and PmAU, using the Venn diagram’s statistical module from the PatternLab V software. 240 proteins were shared between OMVs from both media, 105 proteins were exclusive of PmLB OMVs, and 5 proteins from PmAU OMVs. The proteins mentioned in the text are shown in the picture. (B) Differentially abundant proteins between *P. mirabilis* OMVs obtained from AU and LB. The volcano plot shows the Log2 (*p*-value) on the y-axis and the Log2 (fold-change) on the x-axis. Proteins identified as common to both conditions are represented by a dot in the plot. Blue dots indicate proteins satisfying all statistical criteria and thus are considered as differentially abundant between culture conditions. Selected differential proteins discussed in the text are labeled and information regarding all differential proteins are depicted in Supplementary Table 3.

Using the Venn diagram’s statistical module from the PatternLab V software, we could determine that 47 proteins were exclusively detected in LB and 14 in AU (*p*-value <0.05; Figure 5A; Supplementary Table 3).

In order to analyze proteins present in both conditions, OMVs from *E. coli* in LB broth (EcLB) and AU medium (EcAU), but exhibiting significant differences in their relative abundance, we employed the PatternLab V Pairwise Comparison module. Using this tool, we identified 22 and 12 proteins significantly increased in EcLB and EcAU, respectively (*p*-value <0.05; Figure 5B; Supplementary Table 3).

Within the proteins that were detected only in EcLB OMVs, we identified the fimbrial proteins FimH and FimG, and the flagellar proteins FlgE, FlgK, FlgG, FlgH, FlgL, and FliF. FliC was found in both conditions but increased in LB (Table 3). The lipoproteins Blc, YgdI, YajI and OsmY were detected as exclusive to EcLB OMVs. In addition, OsmE, SlyB, and NlpD were increased in LB, while Lpp showed a relative increased abundance in AU. On the other hand, although OMVs recovered from both cultured conditions express the same membrane porins, some of them were found significantly increased in the LB OMVs, such as OmpA, OmpC and OmpX (Table 3).

**Table 3:** Selected proteins identified in OMVs from UPEC discussed in the text.

Accession (uniprot)	Protein	Description/annotation	Fold-change	*p*-value	Media condition	Subcellular location*
Flagellar proteins						
A0A0H2V8D2	FlgE	Flagellar hook protein	ED	*p* < 0.05	LB	E
A0A0H2V6D5	FlgL	Flagellar hook-associated protein 3	ED	*p* < 0.05	LB	E
A0A0H2V863	FliF	Flagellar M-ring protein	ED	*p* < 0.05	LB	IM
A0A0H2V692	FlgK	Flagellar hook-associated protein 1	ED	*p* < 0.05	LB	E
Q8CW55	FlgH	Flagellar L-ring protein	ED	*p* < 0.05	LB	OM
P0ABX6	FlgG	Flagellar basal-body rod protein	ED	*p* < 0.05	LB	E
A0A0H2VAE3	FliC	Flagellin	4.30	0.008	LB	E
Fimbrial proteins						
A0A0H2VGH2	FimG	FimG protein protein	ED	*p* < 0.05	LB	U
A0A0H2VDU7	FimH	FimH protein	ED	*p* < 0.05	LB	U
Porins proteins						
P0A918	OmpX	Outer membrane protein X	2.20	0.005	LB	OM
A0A0H2V5V4	OmpA	Outer membrane protein A	1.52	0.001	LB	OM
Q8CVW1	OmpC	Outer membrane porin C	1.36	0.001	LB	OM
Lipoproteins						
A0A0H2VF34	Blc	Outer membrane lipoprotein	ED	*p* < 0.05	LB	OM
P65293	YgdI	Uncharacterized lipoprotein	ED	*p* < 0.05	LB	U
A0A0H2V4U4	YajI	Hypothetical lipoprotein	ED	*p* < 0.05	LB	U
A0A0H2V7I8	SlyB	Outer membrane lipoprotein	2.40	0.001	LB	OM
A0A0H2V7T2	OsmE	Osmotically inducible lipoprotein E	1.94	0.009	LB	U
A0A0H2VCF4	NlpD	Lipoprotein	1.81	4.2E-04	LB	OM
P69777	Lpp	Major outer membrane lipoprotein	1.20	0.027	AU	OM
Transport proteins						
A0A0H2V524	FepA	Ferrienterobactin receptor	3.33	3.1E-04	AU	OM

ED, Proteins exclusively detected (*p*-value < 0.05). The media condition can be LB, Luria Bertani Broth; AU, Artificial urine. The subcellular locations are E, Extracellular; IM, Inner membrane; OM, Outer membrane; U, Unknown. *The subcellular localization prediction by Psortb v3. The total number of proteins are in Supplementary Table 3.

Regarding the outer membrane proteins, two of them were found to be increased from EcAU OMVs: Ferrienterobactin receptor (FepA) and Colicin I receptor (CirA).

## 4 Discussion

Outer membrane vesicles (OMVs) are spherical particles released from the outer membrane of Gram-negative bacteria (Beveridge, 1999). The production of OMVs is not only a common physiological mechanism in bacteria but also serves as a response to environmental stress (Kulp and Kuehn, 2010). OMVs are associated with bacterial survival, playing key roles in nutrient acquisition, intra-and interspecific bacterial communication, biofilm formation, defense mechanisms, resistance, and pathogenesis (Kulp and Kuehn, 2010). The composition, function, and quantity of OMVs depend on the specific biogenesis pathways. Studies have shown that OMVs exhibit distinct structures and characteristics depending on their mode of production and the surrounding microenvironment (Kulp and Kuehn, 2010).

The primary mechanism through which Gram-negative bacteria produce OMVs is outer membrane blebbing. As a result, OMVs are enriched in outer membrane proteins, have specific lipid compositions, and differ in cargo molecule content compared to other extracellular vesicles (Orench-Rivera and Kuehn, 2016). In this study, we isolated, characterized, and compared the OMVs produced by *Proteus mirabilis* and *Escherichia coli* cultured in Luria-Bertani (LB) media and artificial urine (AU). Additionally, we conducted a quantitative and comparative proteomic analysis to determine and compare the protein profiles of OMVs derived from both pathogens grown in these two different media.

In a previous study, 619 unique proteins were identified in OMVs from 54 UPEC strains (Wurpel et al., 2015). Wurpel et al. further analyzed the proteome of OMVs from five UPEC reference strains (536, CFT073, F11, UMN026, and UTI89) EDTA-induced in human urine, identifying a distinct set of 173 non-redundant proteins (Wurpel et al., 2016). However, their study provides only an indirect evaluation of the outer membrane composition in UPEC. In contrast, our work evaluates the protein composition of naturally produced OMVs, offering a more direct insight into their biological role.

Outer membrane vesicles (OMVs) are nanoparticles ranging from 20 to 250 nm in diameter (Kulp and Kuehn, 2010), composed of a lipid membrane, lipopolysaccharides (LPS), and periplasmic content. OMVs contain periplasmic and outer membrane proteins, virulence factors, toxins, and genetic material (Pin et al., 2023). Their small size allows them to penetrate tissues typically inaccessible to bacteria (Kuehn and Kesty, 2005). Internalization pathways differ based on OMV size: smaller OMVs (20–100 nm) use caveolin-mediated endocytosis, medium-sized ones (20–250 nm) use clathrin-mediated endocytosis, and larger ones (90–450 nm) rely on macropinocytosis (Wang et al., 2023; O'Donoghue and Krachler, 2016).

In this study, we successfully purified OMVs from *Proteus mirabilis* and *E. coli* grown in LB media and artificial urine (AU). *P. mirabilis* OMVs were significantly larger, with an average diameter over 250 nm, while *E. coli* OMVs were smaller, with diameters under 250 nm. These size variations could influence how OMVs interact with eukaryotic cells. Specifically, the larger size of *P. mirabilis* OMVs may affect the mechanisms of internalization and subsequent immune responses.

Proteomic analysis revealed key OMV proteins, including OmpA, OmpC, and OmpF, common outer membrane protein markers. *P. mirabilis* OMVs showed higher relative abundance of Braun’s lipoprotein (Lpp) in LB-grown cultures, similar to *E. coli* OMVs in AU. Lpp is essential for outer membrane stability by linking it to the peptidoglycan layer (Mathelié-Guinlet et al., 2020). Variations in Lpp abundance and OMV size suggest that protein composition influences OMV structure and function in different environments.

Notably, around 50% of the proteins identified in OMVs were cytoplasmic, suggesting either random cytoplasmic protein incorporation or the production of other vesicle types, such as external-internal membrane vesicles (OIMVs) or cytoplasmic membrane vesicles (CMVs; Toyofuku et al., 2023). Our findings align with reports indicating significant cytoplasmic content in OMVs from Gram-negative bacteria (Kulp and Kuehn, 2010; Charpentier et al., 2023), providing further insight into the complexity of OMV biogenesis.

In contrast to the nutritionally-rich LB media, urine in the bladder represents a high-osmolarity, moderately oxygenated and, iron-limited environment that contains mostly amino acids and small peptides (Brooks and Keevil, 1997). To this must be added that the presence of a constant flow caused by urination poses a challenge to bacteria (Asscher et al., 1966; Stamey and Mihara, 1980). Artificial urine mimics the chemical composition of urine, but lacks the immune system components such as cells, chemokines, interleukins, and microorganisms from the urinary microbiota (Soriano et al., 2009). Comparing the proteomic profile of OMVs in LB and AU can provide evidence regarding the presence of proteins required by the uropathogens in the context of urinary tract infection.

### 4.1 Proteomic content in OMVs from *Proteus mirabilis* 2921

Although the roles of OMVs have been studied extensively, little is known about their production, delivery, and involvement in *P. mirabilis*. This study represents the first report on the proteomic composition of OMVs from *P. mirabilis* under two different culture conditions: LB and artificial urine (AU). We used a *P. mirabilis* strain isolated from a patient with symptomatic UTI (Zunino et al., 2000), known for its high biofilm-forming capacity (Schlapp et al., 2011; Scavone et al., 2023).

Our findings revealed a significant difference in the number of proteins detected in LB compared to AU, showing higher numbers of proteins detected in LB, being this effect more evident in *P. mirabilis* than in *E. coli*. Despite the lower number of proteins detected in AU, those identified were biologically significant, mainly related to iron acquisition—a key response to the iron-depleted conditions in AU, similar to natural urine.

We identified several key OMV markers in *P. mirabilis*-derived OMVs, including OmpA, OmpF, OmpW, TolC, TolB, AcrA, TufB, Pal, and Lpp, confirming the reliability of our vesicle isolation protocol. Flagellar and fimbrial proteins, essential for *P. mirabilis* motility and colonization, were also detected. Notably, MR/P fimbriae components (MrpH, MrpB, MrpE, MrpF, and MrpG) were over-represented in OMVs from LB cultures, while the structural fimbrial subunit MrpA was more abundant in AU, aligning with their roles in urinary tract colonization.

We also identified UCA/NAF fimbriae proteins (PMI0535 and PMI0534) and several PMF fimbriae proteins (PmfA, PmfE, and PmfF), which are critical for bladder and kidney colonization (Zunino et al., 2003). Consistent with previous studies, OmpA and OmpF were more abundant in nutrient-rich LB media (D'Alessandro et al., 2011). Additionally, TonB-dependent receptors (PMI2596, IreA, PMI1548, PMI0363, PMI0233, PMI0409) were over-represented in AU, reflecting the bacterium’s response to iron-restricted conditions.

Among *P. mirabilis* virulence factors, only hemolysin (HpmA) was detected in OMVs from both media conditions. HmpB, which activates HmpA, was exclusively found in AU-derived OMVs, suggesting that OMVs could serve as a mechanism for hemolysin secretion.

### 4.2 Proteomic content in OMVs from *Escherichia coli* 144

In this study, we analyzed the protein content of OMVs produced by a clinical *E. coli* strain grown in Luria-Bertani (LB) broth and artificial urine (AU; Robino et al., 2014). This UPEC strain is known for its ability to form biofilms and invade urothelial cells in the bladder, forming intracellular bacterial communities (González et al., 2017; González et al., 2020). However, unlike other UPEC strains, this isolate does not express typical toxins such as HlyA and CNF1, nor the virulence genes iutA, ibeA, PAI, and fyuA (González et al., 2020). As expected, we did not detect HlyA and CNF1 toxins in the OMVs from *E. coli* 144.

Pathogenic and commensal *E. coli* strains produce OMVs with differing effects. Pathogenic *E. coli* OMVs typically increase pro-inflammatory cytokines, while commensal *E. coli* OMVs have anti-inflammatory effects (Behrouzi et al., 2018). In this sense, it is interesting to highlight that OMV proteins such as OmpA, CirA, and FepA can trigger inflammatory responses from macrophages (Imamiya et al., 2023).

Adhesins play a crucial role in bacterial colonization by mediating adhesion to host tissues. FimH, for example, is an adhesin that enables UPEC to adhere to uroplakin molecules on urothelial cells, making it an important virulence factor (Behzadi and Behzadi, 2016). Blackburn et al. found that FimA was the dominant protein in OMVs from *E. coli* K12 and noted its co-regulation with FliC (Blackburn et al., 2021). In our study, we detected various fimbrial proteins, including FimG and FimH, with their abundance varying based on culture conditions. This suggests that OMVs may act as a delivery mechanism for adhesins, which can also activate various eukaryotic cell mechanisms.

Flagella and type 1 fimbriae are co-regulated and contribute to *E. coli* adhesion and biofilm formation (Blumer et al., 2005; Badea et al., 2009). Flagellum-mediated motility and chemotaxis help UPEC escape immune responses and spread within the urinary tract (Lane et al., 2005). Previous studies have shown downregulation of flagellation-related genes *in vivo* compared to LB growth (Snyder et al., 2004). In this work, FlgL and FlgK were only detected in OMVs from LB, while FliC was more abundant in LB OMVs. Consistently, both identified proteins, type 1 fimbriae (FimH) and flagellin (FliC), activate the immune system via TLR4 and TLR5 receptors (Mossman et al., 2008). Thus, their presence in OMVs is associated with pro-inflammatory cytokine induction.

In urine, the majority of upregulated genes and proteins are involved in iron acquisition, a crucial mechanism for bacteria adaptation to host environments (Alteri and Mobley, 2015). Iron-acquisition systems are vital virulence factors in UPEC (Alteri and Mobley, 2015). Previous studies identified iron-uptake proteins in *E. coli* OMVs, such as the catecholate siderophore receptor Fiu, iron uptake system component EfeO, and ferrichrome-iron receptor FhuA (Rajasekaran et al., 2010; Moeck et al., 1997; Grinter and Lithgow, 2019). OMVs may facilitate the collection of iron-bound siderophores through specific outer membrane receptors. Notably, we observed a significant increase in the relative abundance of the ferrienterobactin receptor FepA in OMVs from UPEC grown in AU. However, despite the high enrichment of TonB-dependent receptors in UPEC during growth in human urine (Alteri and Mobley, 2007), we did not detect TonB proteins in *E. coli* OMVs.

## 5 Conclusion

Both *P. mirabilis* and *E. coli* produce OMVs with distinct characteristics and functions. *P. mirabilis* OMVs are larger and enriched in proteins related to iron acquisition and motility, which are important for its virulence and adaptation to different environments. *E. coli* OMVs, on the other hand, are smaller and contain proteins involved in adhesion, motility, and iron uptake, but lack some typical toxins found in other uropathogenic strains.

The differences in OMV size, protein composition, and functional roles underscore the diversity in OMV-mediated strategies used by these bacteria for survival, adaptation, and pathogenesis in the urinary tract. Understanding the specific characteristics of OMVs in this context, under laboratory conditions mimicking urine, provides valuable insights into the mechanisms of urinary tract infections and could inform targeted therapeutic approaches.

## Data Availability

The mass spectrometry proteomics data have been deposited to the ProteomeXchange Consortium via the PRIDE partner repository with the dataset identifier PXD052681.

## Footnotes

^1^http://www.patternlabforproteomics.org

^2^www.uniprot.org

## References

[ref1] Aguilera L. Toloza L. Gimenez R. Odena A. Oliveira E. Aguilar J.. (2014). Proteomic analysis of outer membrane vesicles from the probiotic strain *Escherichia coli* Nissle 1917. Proteomics 14, 222–229. doi: 10.1002/pmic.201300328, PMID: 24307187

[ref2] Alteri C. J. Mobley H. L. (2007). Quantitative profile of the uropathogenic *Escherichia coli* outer membrane proteome during growth in human urine. Infect. Immun. 75, 2679–2688. doi: 10.1128/IAI.00076-06, PMID: 17513849 PMC1932884

[ref3] Alteri C. J. Mobley H. L. (2015). Metabolism and Fitness of Urinary Tract Pathogens. Microbiol Spectr. doi: 10.1128/microbiolspec.mbp-0016-2015 PMC4510461 26185076

[ref4] Armbruster C. E. Mobley H. L. (2012). Merging mythology and morphology: the multifaceted lifestyle of *Proteus mirabilis*. Nat. Rev. Microbiol. 10, 743–754. doi: 10.1038/nrmicro2890, PMID: 23042564 PMC3621030

[ref5] Armbruster C. E. Mobley H. L. Pearson M. M. (2018). Pathogenesis of *Proteus mirabilis* infection. Eco Sal Plus 8, 10–1128. doi: 10.1128/ecosalplus.esp-0009-2017, PMID: 29424333 PMC5880328

[ref6] Asscher A. W. Sussman M. Waters W. E. Davis R. H. Chick S. (1966). Urine as a medium for bacterial growth. Lancet 288, 1037–1041. doi: 10.1016/S0140-6736(66)92023-X 4162501

[ref7] Badea L. Beatson S. A. Kaparakis M. Ferrero R. L. Hartland E. L. (2009). Secretion of flagellin by the LEE-encoded type III secretion system of enteropathogenic *Escherichia coli*. BMC Microbiol. 9, 1–10. doi: 10.1186/1471-2180-9-30, PMID: 19200386 PMC2647546

[ref8] Behrouzi A. Vaziri F. Rad F. R. Amanzadeh A. Fateh A. Moshiri A.. (2018). Comparative study of pathogenic and non-pathogenic *Escherichia coli* outer membrane vesicles and prediction of host-interactions with TLR signaling pathways. BMC. Res. Notes 11:539. doi: 10.1186/s13104-018-3648-3, PMID: 30068381 PMC6071399

[ref9] Behzadi E. Behzadi P. (2016). The role of toll-like receptors (TLRs) in urinary tract infections (UTIs). Cent European J Urol 69, 404–410. doi: 10.5173/ceju.2016.871, PMID: 28127459 PMC5260452

[ref10] Beveridge T. J. (1999). Structures of gram-negative cell walls and their derived membrane vesicles. J. Bacteriol. 181, 4725–4733. doi: 10.1128/jb.181.16.4725-4733.1999, PMID: 10438737 PMC93954

[ref11] Blackburn S. A. Shepherd M. Robinson G. K. (2021). Reciprocal packaging of the main structural proteins of type 1 fimbriae and flagella in the outer membrane vesicles of “wild type” *Escherichia coli* strains. Ront Microbiol 12:557455. doi: 10.3389/fmicb.2021.557455, PMID: 33643229 PMC7907004

[ref12] Blumer C. Kleefeld A. Lehnen D. Heintz M. Dobrindt U. Nagy G.. (2005). Regulation of type 1 fimbriae synthesis and biofilm formation by the transcriptional regulator LrhA of *Escherichia coli*. Microbiology 151, 3287–3298. doi: 10.1099/mic.0.28098-0, PMID: 16207912

[ref13] Brooks T. Keevil C. (1997). A simple artificial urine for the growth of urinary pathogens. Lett. Appl. Microbiol. 24, 203–206. doi: 10.1046/j.1472-765X.1997.00378.x, PMID: 9080700

[ref14] Chakkour M. Hammoud Z. Farhat S. El Roz A. Ezzeddine Z. Ghssein G. (2024). Overview of *Proteus mirabilis* pathogenicity and virulence. Insights into the role of metals. Front. Microbiol. 15:1383618. doi: 10.3389/fmicb.2024.1383618, PMID: 38646633 PMC11026637

[ref15] Charpentier L. A. Dolben E. F. Hendricks M. R. Hogan D. A. Bomberger J. M. Stanton B. A. (2023). Bacterial outer membrane vesicles and immune modulation of the host. Membranes 13:752. doi: 10.3390/membranes13090752, PMID: 37755174 PMC10536716

[ref16] D'Alessandro B. Lery L. M. S. Von Krüger W. M. A. Lima A. Piccini C. Zunino P. (2011). Proteomic analysis of *Proteus mirabilis* outer membrane proteins reveals differential expression in vivo vs. in vitro conditions. FEMS Immunol. Med. Microbiol. 63, 174–182. doi: 10.1111/j.1574-695X.2011.00839.x, PMID: 22077220

[ref17] Ellis T. N. Kuehn M. J. (2010). Virulence and immunomodulatory roles of bacterial outer membrane vesicles. Microbiol. Mol. Biol. Rev. 74, 81–94. doi: 10.1128/mmbr.00031-09, PMID: 20197500 PMC2832350

[ref18] Foxman B. (2014). Urinary tract infection syndromes: occurrence, recurrence, bacteriology, risk factors, and disease burden. Infect. Dis. Clin. N. Am. 28, 1–13. doi: 10.1016/j.idc.2013.09.003 24484571

[ref19] Gil M. Lima A. Rivera B. Rossello J. Urdániz E. Cascioferro A.. (2019). New substrates and interactors of the mycobacterial serine/threonine protein kinase Pkn G identified by a tailored interactomic approach. J. Proteome 192, 321–333. doi: 10.1016/j.jprot.2018.09.013, PMID: 30267874

[ref20] González M. J. Robino L. Iribarnegaray V. Zunino P. Scavone P. (2017). Effect of different antibiotics on biofilm produced by uropathogenic *Escherichia coli* isolated from children with urinary tract infection. Pathog Dis. 75:053. doi: 10.1093/femspd/ftx053, PMID: 28505288

[ref21] González M. J. Zunino P. Scavone P. Robino L. (2020). Selection of effective antibiotics for uropathogenic *Escherichia coli* intracellular bacteria reduction. Front. Cell. Infect. Microbiol. 10:542755. doi: 10.3389/fcimb.2020.542755, PMID: 33194792 PMC7609437

[ref22] Govindarajan D. K. Kandaswamy K. (2022). Virulence factors of uropathogens and their role in host pathogen interactions. Cell Surface 8:100075. doi: 10.1016/j.tcsw.2022.100075, PMID: 35198842 PMC8841375

[ref23] Govindarajan D. K. Meghanathan Y. Sivaramakrishnan M. Kothandan R. Muthusamy A. Seviour T. W.. (2022). *Enterococcus faecalis* thrives in dual-species biofilm models under iron-rich conditions. Arch. Microbiol. 204:710. doi: 10.1007/s00203-022-03309-7, PMID: 36383258

[ref24] Grinter R. Lithgow T. (2019). The structure of the bacterial iron–catecholate transporter Fiu suggests that it imports substrates via a two-step mechanism. J. Biol. Chem. 294, 19523–19534. doi: 10.1074/jbc.RA119.011018, PMID: 31712312 PMC6926462

[ref25] Hannan T. J. Totsika M. Mansfield K. J. Moore K. H. Schembri M. A. Hultgren S. J. (2012). Host–pathogen checkpoints and population bottlenecks in persistent and intracellular uropathogenic *Escherichia coli* bladder infection. FEMS Microbiol. Rev. 36, 616–648. doi: 10.1111/j.1574-6976.2012.00339.x, PMID: 22404313 PMC3675774

[ref26] Himpsl S. D. Pearson M. M. Arewång C. J. Nusca T. D. Sherman D. H. Mobley H. L. T. (2010). Proteobactin and a yersiniabactin-related siderophore mediate iron acquisition in *Proteus mirabilis*. Mol. Microbiol. 78, 138–157. doi: 10.1111/j.1365-2958.2010.07317.x, PMID: 20923418 PMC2951610

[ref27] Imamiya R. Shinohara A. Yakura D. Yamaguchi T. Ueda K. Oguro A.. (2023). *Escherichia coli*-derived outer membrane vesicles relay inflammatory responses to macrophage-derived exosomes. M Bio 14, e03051–e03022. doi: 10.1128/mbio.03051-22, PMID: 36648227 PMC9973271

[ref28] Kim J. H. Lee J. Park J. Gho Y. S. (2015). Gram-negative and gram-positive bacterial extracellular vesicles. Semin. Cell Dev. Biol. 40, 97–104. doi: 10.1016/j.semcdb.2015.02.006 25704309

[ref29] Kuehn M. J. Kesty N. C. (2005). Bacterial outer membrane vesicles and the host–pathogen interaction. Genes Dev. 19, 2645–2655. doi: 10.1101/gad.1299905 16291643

[ref30] Kulp A. Kuehn M. J. (2010). Biological functions and biogenesis of secreted bacterial outer membrane vesicles. Ann. Rev. Microbiol. 64, 163–184. doi: 10.1146/annurev.micro.091208.073413, PMID: 20825345 PMC3525469

[ref31] Lane M. C. Lockatell V. Monterosso G. Lamphier D. Weinert J. Hebel J. R.. (2005). Role of motility in the colonization of uropathogenic *Escherichia coli* in the urinary tract. Infect. Immun. 73, 7644–7656. doi: 10.1128/iai.73.11.7644-7656.2005, PMID: 16239569 PMC1273871

[ref32] Lee E. Y. Bang J. Y. Park G. W. Choi D. S. Kang J. S. Kim H. J.. (2007). Global proteomic profiling of native outer membrane vesicles derived from *Escherichia coli*. Proteomics 7, 3143–3153. doi: 10.1002/pmic.200700196, PMID: 17787032

[ref33] Lee J. Kim O. Y. Gho Y. S. (2016). Proteomic profiling of gram-negative bacterial outer membrane vesicles: current perspectives. Proteomics Clin. Appl. 10, 897–909. doi: 10.1002/prca.201600032, PMID: 27480505

[ref34] Luna-Pineda V. M. Reyes-Grajeda J. P. Cruz-Córdova A. Saldaña-Ahuactzi Z. Ochoa S. A. Maldonado-Bernal C.. (2016). Dimeric and trimeric fusion proteins generated with fimbrial adhesins of uropathogenic *Escherichia coli*. Front. Cell. Infect. Microbiol. 6:135. doi: 10.3389/fcimb.2016.00135, PMID: 27843814 PMC5087080

[ref35] Magaña G. Harvey C. Taggart C. C. Rodgers A. M. (2024). Bacterial outer membrane vesicles: role in pathogenesis and host-cell interactions. Antibiotics 13:32. doi: 10.3390/antibiotics13010032, PMID: 38247591 PMC10812699

[ref36] Mathelié-Guinlet M. Asmar A. T. Collet J. F. Dufrêne Y. F. (2020). Lipoprotein Lpp regulates the mechanical properties of the *E. coli* cell envelope. Nat. Commun. 11:1789. doi: 10.1038/s41467-020-15489-1, PMID: 32286264 PMC7156740

[ref37] McBroom A. J. Kuehn M. J. (2007). Release of outer membrane vesicles by gram-negative bacteria is a novel envelope stress response. Mol. Microbiol. 63, 545–558. doi: 10.1111/j.1365-2958.2006.05522.x, PMID: 17163978 PMC1868505

[ref38] Mendoza-Barberá E. Merino S. Tomás J. M. (2023). “Bacterial adhesion” in Molecular Medical Microbiology. eds. Tang M. H. Liu D. Sails A. Spearman P. Zhang J.. third ed, Academic press. 359–375.

[ref39] Milo S. Heylen R. A. Glancy J. Williams G. T. Patenall B. L. Hathaway H. J.. (2021). A small-molecular inhibitor against *Proteus mirabilis* urease to treat catheter-associated urinary tract infections. Sci. Rep. 11:3726. doi: 10.1038/s41598-021-83257-2, PMID: 33580163 PMC7881204

[ref40] Moeck G. S. Coulton J. W. Postle K. (1997). Cell envelope signaling in *Escherichia coli*: ligand binding to the ferrichrome-iron receptor FhuA promotes interaction with the energy-transducing protein ton B. J. Biol. Chem. 272, 28391–28397. doi: 10.1074/jbc.272.45.28391 9353297

[ref41] Mossman K. L. Mian M. F. Lauzon N. M. Gyles C. L. Lichty B. Mackenzie R.. (2008). Cutting edge: FimH adhesin of type 1 fimbriae is a novel TLR4 ligand. J. Immunol. 181, 6702–6706. doi: 10.4049/jimmunol.181.10.6702, PMID: 18981086

[ref42] O'Donoghue E. J. Krachler A. M. (2016). Mechanisms of outer membrane vesicle entry into host cells. Cell. Microbiol. 18, 1508–1517. doi: 10.1111/cmi.12655, PMID: 27529760 PMC5091637

[ref43] Orench-Rivera N. Kuehn M. J. (2016). Environmentally controlled bacterial vesicle-mediated export. Cell. Microbiol. 18, 1525–1536. doi: 10.1111/cmi.12676, PMID: 27673272 PMC5308445

[ref44] Pin C. David L. Oswald E. (2023). Modulation of autophagy and cell death by bacterial outer-membrane vesicles. Toxins 15:502. doi: 10.3390/toxins15080502, PMID: 37624259 PMC10467092

[ref45] Rajasekaran M. B. Nilapwar S. Andrews S. C. Watson K. A. (2010). EfeO-cupredoxins: major new members of the cupredoxin superfamily with roles in bacterial iron transport. Biometals 23, 1–17. doi: 10.1007/s10534-009-9262-z, PMID: 19701722

[ref46] Robino L. Scavone P. Araujo L. Algorta G. Zunino P. Pírez M. C.. (2014). Intracellular bacteria in the pathogenesis of *Escherichia coli* urinary tract infection in children. Clin. Infect. Dis. 59, 158–164. doi: 10.1093/cid/ciu634 PMC4650771 25091303

[ref47] Rosen D. A. Hooton T. M. Stamm W. E. Humphrey P. A. Hultgren S. J. (2007). Detection of intracellular bacterial communities in human urinary tract infection. PLoS Med. 4:e329. doi: 10.1371/journal.pmed.0040329, PMID: 18092884 PMC2140087

[ref48] Rossello J. Lima A. Gil M. Rodríguez Duarte J. Correa A. Carvalho P. C.. (2017). The EAL-domain protein fcs R regulates flagella, chemotaxis and type III secretion system in *Pseudomonas aeruginosa* by a phosphodiesterase independent mechanism. Sci. Rep. 7:102. doi: 10.1038/s41598-017-09926-3, PMID: 28860517 PMC5579053

[ref49] Santos M. D. M. Lima D. B. Fischer J. S. G. Clasen M. A. Kurt L. U. Camillo-Andrade A. C.. (2022). Simple, efficient and thorough shotgun proteomic analysis with pattern lab V. Nat. Protoc. 17, 1553–1578. doi: 10.1038/s41596-022-00690-x, PMID: 35411045

[ref50] Scavone P. Iribarnegaray V. González M. J. Navarro N. Caneles-Huerta N. Jara-Wilde J.. (2023). Role of *Proteus mirabilis* flagella in biofilm formation. Rev. Argent. Microbiol. 55, 226–234. doi: 10.1016/j.ram.2023.01.005, PMID: 37076397

[ref51] Schaffer J. N. Pearson M. M. (2015). *Proteus mirabilis* and urinary tract infections. Microbiol. Spectr. 3, 383–433. doi: 10.1128/9781555817404.ch17 PMC4638163 26542036

[ref52] Schlapp G. Scavone P. Zunino P. Härtel S. (2011). Development of 3D architecture of uropathogenic *Proteus mirabilis* batch culture biofilms—a quantitative confocal microscopy approach. J. Microbiol. Methods 87, 234–240. doi: 10.1016/j.mimet.2011.07.021, PMID: 21864585

[ref53] Schwartz D. J. Chen S. L. Hultgren S. J. Seed P. C. (2011). Population dynamics and niche distribution of uropathogenic *Escherichia coli* during acute and chronic urinary tract infection. Infect. Immun. 79, 4250–4259. doi: 10.1128/iai.05339-11, PMID: 21807904 PMC3187256

[ref54] Shanmugasundarasamy T. Govindarajan D. K. Kandaswamy K. (2022). A review on pilus assembly mechanisms in gram-positive and gram-negative bacteria. The cell surface 8:100077. doi: 10.1016/j.tcsw.2022.100077, PMID: 35493982 PMC9046445

[ref55] Snyder J. A. Haugen B. J. Buckles E. L. Lockatell C. V. Johnson D. E. Donnenberg M. S.. (2004). Transcriptome of uropathogenic *Escherichia coli* during urinary tract infection. Infect. Immun. 72, 6373–6381. doi: 10.1128/iai.72.11.6373-6381.2004, PMID: 15501767 PMC523057

[ref56] Soriano F. Huelves L. Naves P. Rodríguez-Cerrato V. del Prado G. Ruiz V.. (2009). In vitro activity of ciprofloxacin, moxifloxacin, vancomycin and erythromycin against planktonic and biofilm forms of *Corynebacterium urealyticum*. J. Antimicrob. Chemother. 63, 353–356. doi: 10.1093/jac/dkn491, PMID: 19056748

[ref57] Stamey T. A. Mihara G. (1980). Observations on the growth of urethral and vaginal bacteria in sterile urine. J. Urol. 124, 461–463. doi: 10.1016/S0022-5347(17)55496-8, PMID: 7420586

[ref58] Stamm W. E. Norrby S. R. (2001). Urinary tract infections: disease panorama and challenges. J. Infect. Dis. 183, S1–S4. doi: 10.1086/318850, PMID: 11171002

[ref59] Subashchandrabose S. Mobley H. L. T. (2015). Virulence and fitness determinants of Uropathogenic *Escherichia coli*. Microbiol Spect 3:ch12. doi: 10.1128/9781555817404.ch12 PMC4566162 26350328

[ref60] Tashiro Y. Hasegawa Y. Shintani M. Takaki K. Ohkuma M. Kimbara K.. (2017). Interaction of bacterial membrane vesicles with specific species and their potential for delivery to target cells. Front. Microbiol. 8:571. doi: 10.3389/fmicb.2017.00571, PMID: 28439261 PMC5383704

[ref61] Toyofuku M. Schild S. Kaparakis-Liaskos M. Eberl L. (2023). Composition and functions of bacterial membrane vesicles. Nat. Rev. Microbiol. 21, 415–430. doi: 10.1038/s41579-023-00875-5 36932221

[ref62] Wang X. Lin S. Wang L. Cao Z. Zhang M. Zhang Y.. (2023). Versatility of bacterial outer membrane vesicles in regulating intestinal homeostasis. Sci. Adv. 9:eade5079. doi: 10.1126/sciadv.ade5079, PMID: 36921043 PMC10017049

[ref63] Wurpel D. J. Moriel D. G. Totsika M. Easton D. M. Schembri M. A. (2015). Comparative analysis of the uropathogenic *Escherichia coli* surface proteome by tandem mass-spectrometry of artificially induced outer membrane vesicles. J. Proteome 115, 93–106. doi: 10.1016/j.jprot.2014.12.005, PMID: 25534882

[ref64] Wurpel D. J. Totsika M. Allsopp L. P. Webb R. I. Moriel D. G. Schembri M. A. (2016). Comparative proteomics of uropathogenic *Escherichia coli* during growth in human urine identify UCA-like (UCL) fimbriae as an adherence factor involved in biofilm formation and binding to uroepithelial cells. J. Proteome 131, 177–189. doi: 10.1016/j.jprot.2015.11.001, PMID: 26546558

[ref65] Yu N. Y. Wagner J. R. Laird M. R. Melli G. Rey S. Lo R.. (2010). PSORTb 3.0: improved protein subcellular localization prediction with refined localization subcategories and predictive capabilities for all prokaryotes. Bioinformatics 26, 1608–1615. doi: 10.1093/bioinformatics/btq249, PMID: 20472543 PMC2887053

[ref66] Zunino P. Geymonat L. Allen A. G. Legnani-Fajardo C. Maskell D. J. (2000). Virulence of a *Proteus Mirabilis* ATF isogenic mutant is not impaired in a mouse model of ascending urinary tract infection. FEMS Immunol. Med. Microbiol. 29, 137–143. doi: 10.1111/j.1574-695X.2000.tb01516.x 11024353

[ref67] Zunino P. Piccini C. Legnani-Fajardo C. (1994). Flagellate and non-flagellate *Proteus mirabilis* in the development of experimental urinary tract infection. Microb. Pathog. 16, 379–385. doi: 10.1006/mpat.1994.1038, PMID: 7815921

[ref68] Zunino P. Sosa V. Allen A. G. Preston A. Geraldine S. Maskell D. J. (2003). *Proteus mirabilis* fimbriae (PMF) are important for both bladder and kidney colonization in mice. Microbiologica 149, 3231–3237. doi: 10.1099/mic.0.26534-0, PMID: 14600235

